# Supplementary figures and images for: Identifying biomarkers for evaluating wound extent and age in the contused muscle of rats using microarray analysis: a pilot study
Source: PeerJ. 2021 Dec 23;9:e12709. doi: 10.7717/peerj.12709 (PMC8710249; doi:10.7717/peerj.12709)

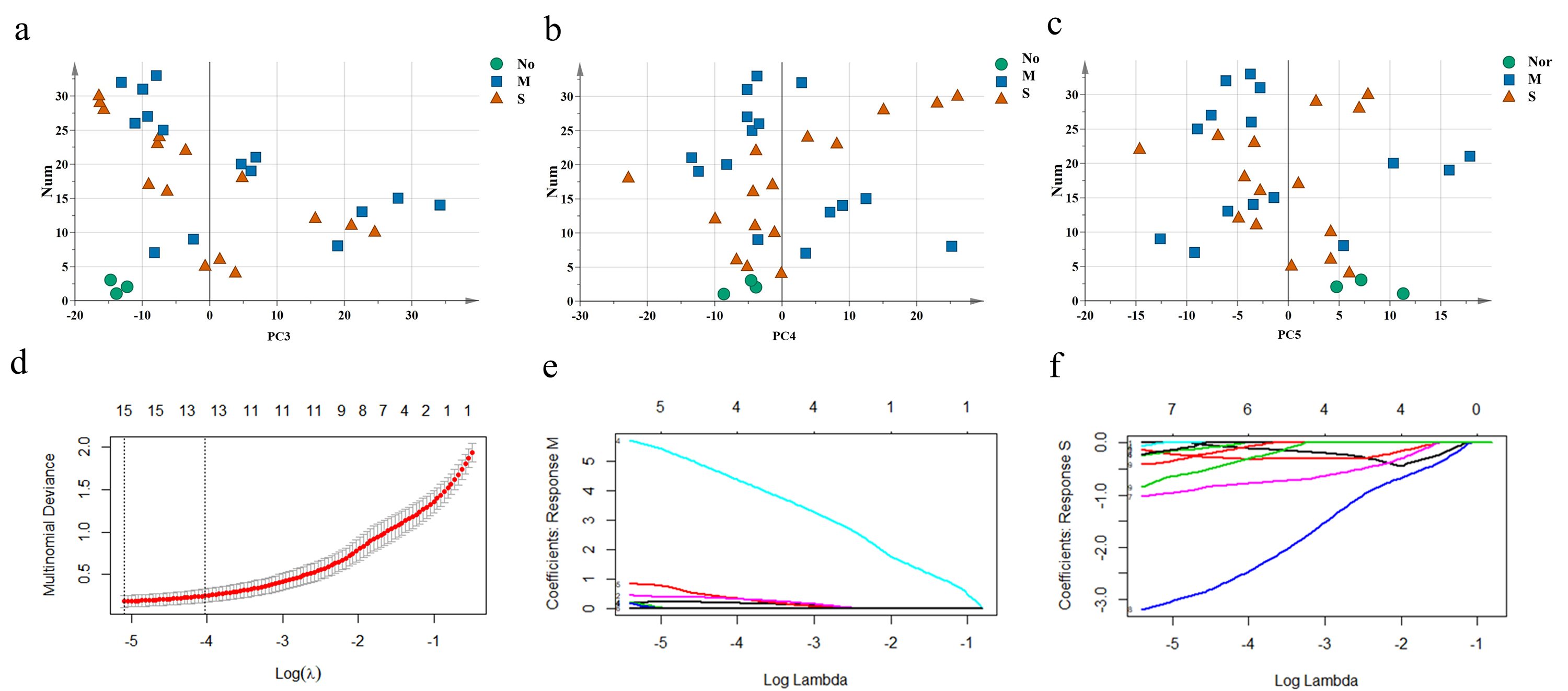

Supplement: Supplemental Information 1 — (A–C) The score plots of principal component (PC)3, PC 4 and PC 5 “Nor” means normal groups (in bluish green); “M” means mild injury groups (in blue); “S” means severe injury groups (in vermilion) . (D) Mean cross-validated errors (red dots) with standard errors (error bar) were used to determine the optimum penalty lambda (λ). The vertical dotted lines at the left of each plot indicates the optimum λ and corresponding descriptor number. (E) LASSO coefficient profiles of the 15 prognostic DEGs for mild contusion groups. (F) LASSO coefficient profiles in severe contusion groups. [file peerj-09-12709-s001.png]
