# Supplementary material for: Identifying biomarkers for evaluating wound extent and age in the contused muscle of rats using microarray analysis: a pilot study
Source: PeerJ. 2021 Dec 23;9:e12709. doi: 10.7717/peerj.12709 (PMC8710249; doi:10.7717/peerj.12709)
Supplement: Supplemental Information 2 [file peerj-09-12709-s002.docx]

**Supplementary Table S1. Primers and probes used for real-time PCR.**

| Gene Symbol | GenBank accession no | Strand | Nucleotide sequence | Reporter | Quencher | Size of PCR products (bp) | Amplification efficiencies |
| --- | --- | --- | --- | --- | --- | --- | --- |
| RPL13 | NM_031101.1 | Sense | TCGTGAGGTGCCCTACAGTTAG |  |  | 107 | 103.2% |
|  |  | Probe | CACACCAAGGTCCGGGCTGGCAG | FAM | BQH |  |  |
|  |  | Antisense | GGTGCGTGCCATTTTCTTGTG |  |  |  |  |
| RPL32 | NM_013226.2 | Sense | ATCTGGCCCTTGAATCTTCTCC |  |  | 115 | 108.1% |
|  |  | Probe | TGTCGATGCCTCTGGGTTTCCGCC | Cy5 | BQH |  |  |
|  |  | Antisense | AGAGGACCAAGAAGTTCATCAGG |  |  |  |  |
| Gpnmb | NM_133298 | Sense | GGCAATATCGTCTATGAGAGGAAC |  |  | 112 | 107.4% |
|  |  | Probe | CATCGTCTGCCCCTGTGGTCCAGT | HEX | BQH |  |  |
|  |  | Antisense | TGGTGTTGTCTTCCCAGTCC |  |  |  |  |
| Rrad | NM_053338 | Sense | CTCAGAGGATGGCGTTTACAAG |  |  | 141 | 104.2% |
|  |  | Probe | CTGCTGCTTCTGCTTCAGGTCCGT | ROX | BQH |  |  |
|  |  | Antisense | ACAGTGATAGAACGGTCATATGTG |  |  |  |  |
| Timp1 | NM_053819 | Sense | TTCGACGCTGTGGGAAATGC |  |  | 147 | 106.4% |
|  |  | Probe | CGGTTCGCCTACACCCCAGCCAT | HEX | BQH |  |  |
|  |  | Antisense | AGTGATGTGCAAATTTCCGTTCC |  |  |  |  |

| Id | Gene | Description | 0h-M | 1h-M | 3h-M | 24h-M | 48h-M | 168h-M | 1h-S | 3h-S | 24h-S | 48h-S | 168h-S |
| --- | --- | --- | --- | --- | --- | --- | --- | --- | --- | --- | --- | --- | --- |
| 17764321 | Apip | APAF1 interacting protein | 6.58982 | 7.00218 | 6.41010 | 6.53391 | 6.53639 | 6.89398 | 6.09673 | 5.96238 | 5.96371 | 5.78248 | 5.92347 |
| 17786640 | Cecr2 | cat eye syndrome chromosome region, candidate 2 | 6.29000 | 6.09017 | 6.06753 | 5.86784 | 5.60200 | 6.51114 | 5.57939 | 5.43773 | 5.60978 | 5.00365 | 5.29721 |
| 17676030 | Fam220a | family with sequence similarity 220, member A | 6.16285 | 6.15070 | 5.97942 | 6.12366 | 5.65423 | 6.41858 | 5.45313 | 5.24581 | 5.48060 | 5.19374 | 5.54241 |
| 17647590 | Gps2 | G protein pathway suppressor 2 | 6.41984 | 7.23244 | 6.66178 | 6.75233 | 7.07781 | 7.36529 | 6.10513 | 5.54218 | 6.16742 | 5.85235 | 6.13984 |
| 17610557 | Hivep2 | human immunodeficiency virus type I enhancer binding protein 2 | 6.59023 | 7.38904 | 7.82575 | 6.76131 | 6.81008 | 7.29327 | 6.58099 | 6.35087 | 6.37900 | 6.00200 | 6.75328 |
| 17808508 | Mllt3 | myeloid/lymphoid or mixed-lineage leukemia; translocated to, 3 | 7.50503 | 8.78653 | 8.40128 | 7.83790 | 8.03571 | 8.68215 | 7.88484 | 7.31542 | 7.45344 | 6.53136 | 7.23523 |
| 17664070 | Myadml2 | myeloid-associated differentiation marker-like 2 | 6.24231 | 5.85764 | 5.57971 | 5.27678 | 5.58665 | 6.13418 | 5.45919 | 5.10989 | 5.18440 | 4.83969 | 4.95169 |
| 17657398 | Ntn1 | netrin 1 | 4.36730 | 5.28780 | 5.11085 | 4.69772 | 4.60368 | 5.21248 | 4.26515 | 4.00993 | 3.55299 | 3.91146 | 4.22362 |
| 17698613 | Oxa1l | oxidase (cytochrome c) assembly 1-like | 8.98019 | 8.68616 | 8.31578 | 8.18575 | 8.30488 | 8.78181 | 8.26708 | 7.88003 | 8.14474 | 7.70697 | 8.41759 |
| 17851110 | Spa17 | sperm autoantigenic protein 17 | 5.00510 | 6.05244 | 5.49995 | 5.22877 | 5.17050 | 6.25798 | 5.19877 | 4.92877 | 4.52764 | 4.59438 | 4.89458 |
| 17677169 | Tmem248 | transmembrane protein 248 | 7.39611 | 7.78705 | 7.38960 | 7.37343 | 7.58775 | 8.11332 | 6.78837 | 6.50112 | 6.92535 | 6.81421 | 6.87743 |
| 17621076 | Trim72 | tripartite motif containing 72, E3 ubiquitin protein ligase | 8.02347 | 7.92666 | 8.28858 | 7.75800 | 8.13011 | 8.13329 | 7.48033 | 7.24495 | 7.27439 | 6.93748 | 7.52173 |
| 17823741 | Ttc7b | tetratricopeptide repeat domain 7B | 8.26405 | 8.00371 | 7.71347 | 7.30721 | 7.56567 | 8.16895 | 7.57663 | 7.25544 | 7.20753 | 6.90803 | 7.44125 |
| 17643839 | Ubald1 | UBA-like domain containing 1 | 5.71426 | 6.86514 | 6.45475 | 6.53813 | 6.49669 | 6.86534 | 5.48089 | 5.01816 | 5.28841 | 5.23901 | 5.48050 |
| 17835977 | Yeats4 | YEATS domain containing 4 | 7.42851 | 8.28913 | 7.98431 | 7.69339 | 7.63114 | 8.43022 | 7.51386 | 7.15742 | 7.32085 | 7.01851 | 6.88805 |

**Supplementary Table S2. Results of fifteen markers for wound degree estimation.**

**Supplementary Table S3. Top 60 provided genes for mild wound estimation.**

| Gene symbol | Description | Gene symbol | Description |
| --- | --- | --- | --- |
| Spp1 | secreted phosphoprotein 1 | Pfkfb1 | 6-phosphofructo-2-kinase/fructose-2,6-biphosphatase 1 |
| Cd8a | CD8a molecule | Mss51 | MSS51 mitochondrial translational activator |
| Prg4 | proteoglycan 4 | Adipoq | adiponectin, C1Q and collagen domain containing |
| Gpnmb | glycoprotein (transmembrane) nmb | Trem1 | triggering receptor expressed on myeloid cells 1 |
| Cd163 | CD163 molecule | Il1rn | interleukin 1 receptor antagonist |
| Lgmn | legumain | Ccl2 | chemokine (C-C motif) ligand 2 |
| Msr1 | macrophage scavenger receptor 1 | Clec7a | C-type lectin domain family 7, member A |
| Il18 | interleukin 18 | Ccr1 | chemokine (C-C motif) receptor 1 |
| Clec4a1 | C-type lectin domain family 4, member A1 | Csf2rb | colony stimulating factor 2 receptor, beta, low-affinity (granulocyte-macrophage) |
| Apobec1 | apolipoprotein B mRNA editing enzyme, catalytic polypeptide 1 | Csf3r | colony stimulating factor 3 receptor (granulocyte) |
| Mrc1 | mannose receptor, C type 1 | Cxcr2 | chemokine (C-X-C motif) receptor 2 |
| Ifitm1 | interferon induced transmembrane protein 1 | S100a8 | S100 calcium binding protein A8 |
| S100a4 | S100 calcium-binding protein A4 | S100a9 | S100 calcium binding protein A9 |
| Hba-a1 | hemoglobin alpha, adult chain 1 | Sell | selectin L |
| Il6 | interleukin 6 | Clec4d | C-type lectin domain family 4, member D |
| Mmp8 | matrix metallopeptidase 8 | Serpine1 | serpin peptidase inhibitor, clade E (nexin, plasminogen activator inhibitor type 1), member 1 |
| Cxcl2 | chemokine (C-X-C motif) ligand 2 | Rrad | Ras-related associated with diabetes |
| Il1b | interleukin 1 beta | Hmox1 | heme oxygenase 1 |
| Nos2 | nitric oxide synthase 2, inducible | Tubb6 | tubulin, beta 6 class V |
| Socs3 | suppressor of cytokine signaling 3 | Pdpn | podoplanin |
| Cxcl6 | chemokine (C-X-C motif) ligand 6 | Mt2A | metallothionein 2A |
| Egr1 | early growth response 1 | Ccl7 | chemokine (C-C motif) ligand 7 |
| Sln | sarcolipin | Lilrb4 | leukocyte immunoglobulin-like receptor, subfamily B, member 4 |
| Tnni1 | troponin I type 1 (skeletal, slow) | Pla2g7 | phospholipase A2, group VII (platelet-activating factor acetylhydrolase, plasma) |
| Myoz2 | myozenin 2 | Timp1 | TIMP metallopeptidase inhibitor 1 |
| Tnnc1 | troponin C type 1 (slow) | Lyz2 | lysozyme 2 |
| Myl3 | myosin, light chain 3, alkali; ventricular, skeletal, slow | Cd53 | Cd53 molecule |
| Myh7 | myosin, heavy chain 7, cardiac muscle, beta | PVR | poliovirus receptor |
| Tnnt1 | troponin T type 1 (skeletal, slow) | Serpina3n | serine (or cysteine) peptidase inhibitor, clade A, member 3N |
| Ky | kyphoscoliosis peptidase | Ankrd1 | ankyrin repeat domain 1 |

| Gene symbol | Description | Gene symbol | Description |
| --- | --- | --- | --- |
| Spp1 | secreted phosphoprotein 1 | Ccl7 | chemokine (C-C motif) ligand 7 |
| Gpnmb | glycoprotein (transmembrane) nmb | Pf4 | platelet factor 4 |
| Lilrb4 | leukocyte immunoglobulin-like receptor, subfamily B, member 4 | Clec7a | C-type lectin domain family 7, member A |
| Hmox1 | heme oxygenase 1 | Ccr1 | chemokine (C-C motif) receptor 1 |
| Clec4a3 | C-type lectin domain family 4, member A3 | Il1rn | interleukin 1 receptor antagonist |
| S100a4 | S100 calcium-binding protein A4 | Vcan | versican |
| Cybb | cytochrome b-245, beta polypeptide | Ifitm1 | interferon induced transmembrane protein 1 |
| Ccl2 | chemokine (C-C motif) ligand 2 | Evi2b | ecotropic viral integration site 2B |
| Apoe | apolipoprotein E | Anpep | alanyl (membrane) aminopeptidase |
| Lgmn | legumain | Mrc1 | mannose receptor, C type 1 |
| Cd68 | Cd68 molecule | Pdpn | podoplanin |
| Hba-a1 | hemoglobin alpha, adult chain 1 | Myl3 | myosin, light chain 3, alkali; ventricular, skeletal, slow |
| Clec4a1 | C-type lectin domain family 4, member A1 | Pltp | phospholipid transfer protein |
| Lgals3 | lectin, galactoside-binding, soluble, 3 | Msr1 | macrophage scavenger receptor 1 |
| Apobec1 | apolipoprotein B mRNA editing enzyme, catalytic polypeptide 1 | Gpr34 | G protein-coupled receptor 34 |
| Slpi | secretory leukocyte peptidase inhibitor | Cd163 | CD163 molecule |
| Ankrd1 | ankyrin repeat domain 1 | Myo5a | myosin VA |
| Fos | FBJ osteosarcoma oncogene | Lum | lumican |
| Cd53 | Cd53 molecule | Rgs18 | regulator of G-protein signaling 18 |
| Ptprc | protein tyrosine phosphatase, receptor type, C | Il6 | interleukin 6 |
| Lyz2 | lysozyme 2 | Tlr7 | toll-like receptor 7 |
| Mt2A | metallothionein 2A | Clec4d | C-type lectin domain family 4, member D |
| Timp1 | TIMP metallopeptidase inhibitor 1 | Tyrobp | Tyro protein tyrosine kinase binding protein |
| Fcgr1a | Fc fragment of IgG, high affinity Ia, receptor (CD64) | Csf1r | colony stimulating factor 1 receptor |
| Akr1b8 | aldo-keto reductase family 1, member B8 | Pla2g7 | phospholipase A2, group VII (platelet-activating factor acetylhydrolase, plasma) |
| Fcer1g | Fc fragment of IgE, high affinity I, receptor for; gamma polypeptide | Grn | granulin |
| Ly49si1; LOC690045 | immunoreceptor Ly49si1; similar to immunoreceptor Ly49si1 [Source:RGD Symbol;Acc:1585167] | Nr4a1 | nuclear receptor subfamily 4, group A, member 1 |
| Egr1 | early growth response 1 | Ms4a6bl; Ms4a6b | membrane-spanning 4-domains, subfamily A, member 6B-like; membrane-spanning 4-domains, subfamily A, member 6B |
| Lcp1 | lymphocyte cytosolic protein 1 | Slfn4 | schlafen 4 |
| C3ar1 | complement component 3a receptor 1 | Rrad | Ras-related associated with diabetes |

**Supplementary Table S4. Top 60 provided genes for severe wound estimation.**

**Supplementary Table S5. Expression levels of Gpnmb, Rrad and Timp.**

| Group | Rrad | Gpnmb | Timp1 |
| --- | --- | --- | --- |
| Ctrl | 1±0.32 | 1±0.29 | 1±0.13 |
| 4h | 52.86±24.38* (p<0.001) | 1.82±0.54 | 4±0.35* (p<0.001) |
| 8h | 27.77±12.47* (p=0.007) | 2.2±0.6 | 6.61±0.98* (p<0.001) |
| 12h | 78.91±34.2* (p<0.001) | 9.11±1.22* (p<0.001) | 5.78±1.17* (p<0.001) |
| 16h | 56.92±12.96* (p<0.001) | 12.66±4.4* (p<0.001) | 5.75±0.86* (p<0.001) |
| 20h | 32.81±9.47* (p=0.001) | 15.32±6.2* (p<0.001) | 5.14±0.82* (p<0.001) |
| 24h | 25.22±5.37* (p=0.011) | 21.75±3.78* (p<0.001) | 4.52±0.75* (p<0.001) |

Note: Data are presented as means ± SD.n = 6 per group. * P was were considered to indicate statistical significance compared with control group.

**Supplementary Table S6. Fisher discriminant analysis of the mild and severe contusion groups.**

| Groups |  |  | Predicted Group Membership | | | Total |
| --- | --- | --- | --- | --- | --- | --- |
|  |  |  | 0 | 1 | 2 |  |
| Original^a^ | Count | 0 | 3 | 0 | 0 | 3 |
|  |  | 1 | 0 | 15 | 0 | 15 |
|  |  | 2 | 0 | 0 | 15 | 15 |
|  | % | 0 | 100.0 | .0 | .0 | 100.0 |
|  |  | 1 | .0 | 100.0 | .0 | 100.0 |
|  |  | 2 | .0 | .0 | 100.0 | 100.0 |
| Cross-validated^b,c^ | Count | 0 | 2 | 0 | 1 | 3 |
|  |  | 1 | 0 | 15 | 0 | 15 |
|  |  | 2 | 1 | 1 | 13 | 15 |
|  | % | 0 | 66.7 | .0 | 33.3 | 100.0 |
|  |  | 1 | .0 | 100.0 | .0 | 100.0 |
|  |  | 2 | 6.7 | 6.7 | 86.7 | 100.0 |

a. 100.0% of original grouped cases correctly classified.

b. Cross validation is done only for those cases in the analysis. In cross validation, each case is classified by the functions derived from all cases other than that case.

c. 90.9% of cross-validated grouped cases correctly classified.

**Supplementary Table S7. Fisher discriminant analysis of the estimation of mild injury.**

| Groups |  |  | Predicted Group Membership | | | | | | Total |
| --- | --- | --- | --- | --- | --- | --- | --- | --- | --- |
|  |  |  | 0 | 1 | 2 | 3 | 4 | 5 |  |
| Original | Count | 0 | 3 | 0 | 0 | 0 | 0 | 0 | 3 |
|  |  | 1 | 0 | 3 | 0 | 0 | 0 | 0 | 3 |
|  |  | 2 | 0 | 0 | 3 | 0 | 0 | 0 | 3 |
|  |  | 3 | 0 | 0 | 0 | 3 | 0 | 0 | 3 |
|  |  | 4 | 0 | 0 | 0 | 0 | 3 | 0 | 3 |
|  |  | 5 | 0 | 0 | 0 | 0 | 0 | 3 | 3 |
|  | % | 0 | 100.0 | .0 | .0 | .0 | .0 | .0 | 100.0 |
|  |  | 1 | .0 | 100.0 | .0 | .0 | .0 | .0 | 100.0 |
|  |  | 2 | .0 | .0 | 100.0 | .0 | .0 | .0 | 100.0 |
|  |  | 3 | .0 | .0 | .0 | 100.0 | .0 | .0 | 100.0 |
|  |  | 4 | .0 | .0 | .0 | .0 | 100.0 | .0 | 100.0 |
|  |  | 5 | .0 | .0 | .0 | .0 | .0 | 100.0 | 100.0 |
| Cross-validated | Count | 0 | 3 | 0 | 0 | 0 | 0 | 0 | 3 |
|  |  | 1 | 0 | 3 | 0 | 0 | 0 | 0 | 3 |
|  |  | 2 | 0 | 0 | 3 | 0 | 0 | 0 | 3 |
|  |  | 3 | 0 | 0 | 0 | 3 | 0 | 0 | 3 |
|  |  | 4 | 0 | 0 | 0 | 0 | 3 | 0 | 3 |
|  |  | 5 | 0 | 0 | 0 | 0 | 1 | 2 | 3 |
|  | % | 0 | 100.0 | .0 | .0 | .0 | .0 | .0 | 100.0 |
|  |  | 1 | .0 | 100.0 | .0 | .0 | .0 | .0 | 100.0 |
|  |  | 2 | .0 | .0 | 100.0 | .0 | .0 | .0 | 100.0 |
|  |  | 3 | .0 | .0 | .0 | 100.0 | .0 | .0 | 100.0 |
|  |  | 4 | .0 | .0 | .0 | .0 | 100.0 | .0 | 100.0 |
|  |  | 5 | .0 | .0 | .0 | .0 | 33.3 | 66.7 | 100.0 |

a. 100.0% of original grouped cases correctly classified.

b. Cross validation is done only for those cases in the analysis. In cross validation, each case is classified by the functions derived from all cases other than that case.

94.4% of cross-validated grouped cases correctly classified.

**Supplementary Table S8. Fisher discriminant analysis of the estimation of severe injury.**

| Groups |  |  | Predicted Group Membership | | | | | | Total |
| --- | --- | --- | --- | --- | --- | --- | --- | --- | --- |
|  |  |  | 0 | 1 | 2 | 3 | 4 | 5 |  |
| Original | Count | 0 | 3 | 0 | 0 | 0 | 0 | 0 | 3 |
|  |  | 1 | 0 | 3 | 0 | 0 | 0 | 0 | 3 |
|  |  | 2 | 0 | 0 | 3 | 0 | 0 | 0 | 3 |
|  |  | 3 | 0 | 0 | 0 | 3 | 0 | 0 | 3 |
|  |  | 4 | 0 | 0 | 0 | 0 | 3 | 0 | 3 |
|  |  | 5 | 0 | 0 | 0 | 0 | 0 | 3 | 3 |
|  | % | 0 | 100.0 | .0 | .0 | .0 | .0 | .0 | 100.0 |
|  |  | 1 | .0 | 100.0 | .0 | .0 | .0 | .0 | 100.0 |
|  |  | 2 | .0 | .0 | 100.0 | .0 | .0 | .0 | 100.0 |
|  |  | 3 | .0 | .0 | .0 | 100.0 | .0 | .0 | 100.0 |
|  |  | 4 | .0 | .0 | .0 | .0 | 100.0 | .0 | 100.0 |
|  |  | 5 | .0 | .0 | .0 | .0 | .0 | 100.0 | 100.0 |
| Cross-validated | Count | 0 | 3 | 0 | 0 | 0 | 0 | 0 | 3 |
|  |  | 1 | 0 | 3 | 0 | 0 | 0 | 0 | 3 |
|  |  | 2 | 0 | 0 | 3 | 0 | 0 | 0 | 3 |
|  |  | 3 | 0 | 0 | 0 | 3 | 0 | 0 | 3 |
|  |  | 4 | 0 | 0 | 0 | 0 | 3 | 0 | 3 |
|  |  | 5 | 0 | 0 | 0 | 0 | 1 | 2 | 3 |
|  | % | 0 | 100.0 | .0 | .0 | .0 | .0 | .0 | 100.0 |
|  |  | 1 | .0 | 100.0 | .0 | .0 | .0 | .0 | 100.0 |
|  |  | 2 | .0 | .0 | 100.0 | .0 | .0 | .0 | 100.0 |
|  |  | 3 | .0 | .0 | .0 | 100.0 | .0 | .0 | 100.0 |
|  |  | 4 | .0 | .0 | .0 | .0 | 100.0 | .0 | 100.0 |
|  |  | 5 | .0 | .0 | .0 | .0 | .0 | 100.0 | 100.0 |

a. 100.0% of original grouped cases correctly classified.

b. Cross validation is done only for those cases in the analysis. In cross validation, each case is classified by the functions derived from all cases other than that case.

c. 100.0% of cross-validated grouped cases correctly classified.
