# Supplementary material for: Identifying biomarkers for evaluating wound extent and age in the contused muscle of rats using microarray analysis: a pilot study
Source: PeerJ. 2021 Dec 23;9:e12709. doi: 10.7717/peerj.12709 (PMC8710249; doi:10.7717/peerj.12709)
Supplement: Supplemental Information 5 [file peerj-09-12709-s005.doc]

Application Format for Ethical Approval of the Ethics Committee of Shanxi Medical University

| Project Support Organization：Shanxi Medical University | | Approval number：2019sll002 |
| --- | --- | --- |
| Project name：Construction of multi index fluorescence detection system after injury and its forensic application | | Beginning and ending time of the project:  2019-7.1-2021.6.31 |
| Project types：A：new drugs clinical trials B: new device clinical trials C: √ animal experiments  D：√Human samples collection | | |
| Personal information of Principal Investigator | | |
| Name: Jun-hong Sun | Academic degree：Doctor | Department of forensic pathology, School of Forensic Medicine |
| Tel.No.：13934581996  E-mail: [Sunjunhong146@163.com](mailto:Sunjunhong146@163.com) | | Address：98 University Street, Yuci District, Jinzhong 030604, Shanxi, People’s Republic of China |
| Major research area: Forensic pathology and skeletal muscle injury | | |
| Declaration：  All the above contents (including the attached materials) are true. If approved, I will carry out the study in strict accordance with the provided plan and abide the relevant regulations of the Ethics Committee of Shanxi Medical University  Declarant: Jun-hong Sun Date：2019.7.24 Department：School of Forensic Medicine | | |
| Approval opinion of the Ethics Committee of Shanxi Medical University    Agree to carry out the experiment according to the project content  Chief of the Committee Date 2019.7.29 the Ethics Committee of Shanxi Medical University | | |
