# Supplementary material for: Identifying biomarkers for evaluating wound extent and age in the contused muscle of rats using microarray analysis: a pilot study
Source: PeerJ. 2021 Dec 23;9:e12709. doi: 10.7717/peerj.12709 (PMC8710249; doi:10.7717/peerj.12709)
Supplement: Supplemental Information 6 [file peerj-09-12709-s006.doc]

**Miame Checklist**

# **1. Experiment Design**

Type of Experiment: control group vs contusion groups; time course

Experimental variables: Three groups of rats: control group (without contusion, n=3)

mild contusion group (n=15)

severe contusion group (n=15)

For mild and severe contusion groups were furtherdivided into five subgroups (1, 3, 24, 48, and 168 h post-injury; n = 3 per subgroup).

Hybridization array: Rat Gene 2.0 ST Arrays, Affymetrix®

Tissue used for slide: Skeletal muscle

other variables: In total 33 Sprague Dawley male rats, 10-12 weeks old, weighing 250-300 g,

# **2. Array design**

Array series: Amplified and biotinylated sense-strand DNA targets of Whole-transcripts.

Deconvoluted spot list with gene names: Deconvoluted spot list was available in the [supplementary](javascript:;) files named RaGene-2_0-st-v1.na36.rn5.transcript.

Array type: The probes on the arrays have been selected to be distributed throughout the entire length of each transcript of rats. The hundreds of thousands of probes on each gene ST array is designed to every exon of every transcript represented on the array. Transcript coverage was median of 22 probes per gene.

Slide type (and coating): light-controlled in situ synthesis of DNA microarrays; glass substrate

# **3. Samples**

Source of the sample: For contusion group (mild and severe), skeletal muscle from the wound site of the right posterior limb at 1-, 3-, 24-, 48-, 168-h post-contusion (n=3).

For control group, skeletal muscle from same site as wound site of the right posterior limb

Skeletal muscle contusion model:

For mild contusion groups (n=15): a 500-g counterpoise fell freely from a height of 15 cm cross a clear Lucite guide tube onto the thigh muscles of the right posterior limb and the potential energy of contusion was about 1.46 J/cm2 (the formula: Ep=mgh).

For severe contusion groups (n=15): a 500-g counterpoise fell freely from a height of 50 cm, and the site and the potential energy of contusion was about 2.58J/cm2 (the formula: Ep=mgh).

Sample extraction protocol used: Total RNA was extracted from the skeletal muscle specimens (approximately 50 mg each) using RNAiso Plus 9108 (Takara Bio, Shiga, Japan), following the manufacturer’s instructions.

Labelling protocol used: In conjunction with the Affymetrix® GeneChip® WT Terminal Labeling Kit, the Ambion® WT Expression Kit is designed to generate amplified and biotinylated sensestrand DNA targets from the entire expressed genome.

# **4. Hybridizations**

Hybridization protocol: Hybridizations were performed for 17 hours at at 45°C and 60 rpm using GeneChip Hybridization Oven 645.

Washing Protocol: A standard series of high stringency washes were performed using GeneChip Fluidics Station 450.

# **5. Measurement**

Scanning and software: GeneChip Scanner 3000 7G (Packard Bioscience) was used and it was controlled by the software named GeneChip Command Console Software.

Data Files: 33 raw data files in CEL file

Type of Data: Raw data in in CEL file format

Data Transformation: Raw data of CEL files were normalized and transformed in to CHP files by RMA algorithm using Transcriptome Analysis Console software (version 4.0.1; Affymetrix®).

# **6. Normalization controls**

For comparison, one-way analysis of variance of the contusion and control groups were performed. mRNAs with a >two-fold change in mean expression compared with the control group, (P<0.05, false discovery rate [FDR] < 0.05) were considered as differentially expressed genes.
